# Longitudinal effects of compassion training on emotion regulation and prosocial moral development among adolescents

**DOI:** 10.3389/fpsyg.2026.1969277

**Published:** 2026-10-13

**Authors:** Yutong Sun

**Affiliations:** School of Education Science, Nanjing Normal University, Nanjing, Jiangsu, China

**Keywords:** adolescence, cluster-randomized controlled trial, compassion training, emotion regulation, latent growth modeling, longitudinal analysis, prosocial moral development, RI-CLPM

## Abstract

This cluster-randomized trial compared eight weeks of compassion training with an intensity-matched relaxation program in 876 adolescents from 34 classes in four Chinese secondary schools. Assessments occurred at baseline, post-intervention, and 3- and 12-month follow-ups. Three-level models showed greater baseline-to-follow-up change under compassion training at all post-baseline waves for emotion regulation (ER) and prosocial moral development (PMD; all *p* < 0.001). At 12 months, differential change was 2.203 points for ER (approximate 95% CI 1.266–3.140; baseline-SD-standardized effect 0.317) and 2.649 points for PMD (1.526–3.772; standardized effect 0.278). Piecewise models showed larger intervention-period gains followed by partial attenuation, supporting H1 and H2 and partially supporting H3. In the compassion condition, ER predicted subsequent PMD across all three intervals, whereas the reverse paths were significant in the first two intervals only. Between-condition Wald tests were nonsignificant for all six cross-lagged parameters (*p* = 0.067–0.648), providing no support for H4. Retention at 12 months was 59.6%. Differential changes remained positive and significant across delta-adjusted sensitivity scenarios, although their magnitudes decreased as departures from MAR increased. Compassion training was associated with sustained relative advantages in this sample, without evidence of stronger reciprocal coupling than relaxation training.

## 1 Introduction

During adolescence, emotion regulation and moral cognition appear to mature along intertwined pathways, and early patterns of prosocial engagement appear to take shape. Among the interventions proposed to support this development, compassion training has attracted growing interest as a possible complement to established approaches such as mindfulness-based programs. At least in principle, compassion training is distinguished by its explicit focus on the affective-motivational link between noticing another’s distress and being moved to act on it. If supported, this mechanism could make compassion training particularly relevant during a period in which emotional and moral capacities are still developing (Maratos et al., 2024; Vaish and Grossmann, 2024; Vieten et al., 2024; Aguilera et al., 2025).

This conceptual appeal, however, raises a more pressing practical concern. Schools and clinicians increasingly seek interventions that can be delivered broadly without singling out particular students, rather than interventions reserved for those already identified as struggling. Without stronger longitudinal evidence, compassion training risks remaining in an evidentiary gray zone: theoretically plausible and occasionally supported by short-term findings, but not sufficiently validated to distinguish it confidently from more generic classroom activities that share little of its intended mechanism (Hayes et al., 2025).

The existing literature provides only partial answers. Most compassion-training studies rely on single- or two-timepoint designs, which may capture change immediately after an intervention but are poorly suited to distinguishing temporary shifts from more lasting development. Even when longitudinal data have been collected, researchers have seldom clearly distinguished stable between-person differences from the finer-grained within-person fluctuations that more directly reflect developmental processes. The use of inert comparison groups—wait-list or no-contact controls—is also common, leaving open the possibility that any observed benefit reflects structured attention or the general experience of participating in a class rather than a process specific to compassion cultivation (Kander et al., 2024; Neuenschwander and von Gunten, 2025; Roberts et al., 2026).

A related gap concerns the relationship between emotion regulation and prosocial moral development. Prior work has generally treated these constructs as separate, parallel outcomes rather than as processes that may unfold together over time. Consequently, the more fundamental question of directionality remains largely unresolved: whether gains in emotion regulation tend to precede growth in moral engagement, whether the reverse sequence is more accurate, or whether the two develop in tandem. This uncertainty reflects, at least in part, a shortage of designs with sufficient temporal resolution and analytic approaches, such as random-intercept cross-lagged modeling, capable of isolating within-person ordering from stable trait-level covariation (Hamaker et al., 2015; van Meegen et al., 2024; Li et al., 2025b).

Taken together, the literature provides limited integrated evidence from studies that track intervention effects across both the active training period and an extended follow-up, reduce nonspecific classroom effects through an active, intensity-matched control, and examine the directional interplay between emotional and moral development within individuals rather than only their separate group-level trajectories (Juul et al., 2025; Roberts et al., 2026).

The present study addresses this gap through a cluster-randomized controlled trial comparing an 8-week compassion-training curriculum with an intensity-matched relaxation-training control. Outcomes were assessed at four time points—baseline, immediate posttest, and 3- and 12-month follow-ups—and examined using a tiered analytic strategy: multilevel modeling to estimate overall intervention effects, piecewise latent growth curve modeling to characterize the pattern of change during the intervention and follow-up phases, and random-intercept cross-lagged panel modeling to test bidirectional within-person prediction between emotion regulation and prosocial moral development.

The design combines a genuinely active comparison condition, a four-wave longitudinal structure, and an analytic framework capable of separating trait-level stability from developmental processes. This combination is intended to provide a tentative but better-grounded account of whether compassion training yields benefits beyond ordinary classroom participation and how emotional and moral development may coevolve during adolescence. These findings may, in turn, offer modest guidance for both the theoretical understanding of compassion-based mechanisms and the practical question of whether such programs warrant broader school-based investment (Ritgens et al., 2024; Ward-Seidel et al., 2025).

## 2 Literature review

### 2.1 Theoretical foundations

Compassion has increasingly been distinguished from empathy and sympathy within contemplative science and developmental psychology. While empathy denotes the capacity to resonate affectively with another’s internal state, compassion is conceptualized as a motivational construct comprising four interrelated components: awareness of suffering, emotional resonance with that suffering, a cognitive appraisal of shared human vulnerability, and a behavioral or motivational orientation toward alleviating it. This motivational specificity distinguishes compassion training from purely attentional or awareness-based practices such as mindfulness, positioning it as an intervention that targets the affiliative and prosocial dimensions of emotional experience rather than attentional regulation alone (Vaish and Grossmann, 2024; Vieten et al., 2024).

The present curriculum used an integrative school-based framework informed by Compassion Focused Therapy (CFT) and Compassionate Mind Training (CMT), together with self-compassion, developmental social–emotional learning, and adolescent perspective-taking. Gilbert’s evolutionary and biopsychosocial account links compassion to caregiving motivation and the regulation of threat, drive, and soothing or safeness systems (Gilbert, 2014). Self-compassion contributes kind, balanced responses to personal difficulty and recognition of shared humanity (Neff, 2003). Within this curriculum, compassion was operationalized as recognizing suffering in oneself or others and developing the motivation and skills to respond helpfully. Emotion labeling supported recognition of distress, compassionate self-talk addressed self-criticism, and perspective-taking, imagery, and role-play connected concern for others with supportive action.

These principles were adapted to classroom learning through emotion-awareness tasks, discussion, scenario work, and home practice. The program integrated these approaches within an educational curriculum adapted for adolescents.

Within the process-model tradition, emotion regulation is understood as the set of strategies that individuals use to modulate the occurrence, intensity, duration, and expression of emotional experience. These include antecedent-focused strategies, such as cognitive reappraisal, and response-focused strategies, such as suppression. Adolescence is characterized by pronounced neurodevelopmental asynchrony between limbic reactivity and prefrontal regulatory capacity, making it both a period of heightened emotional vulnerability and a window of substantial plasticity for acquiring regulatory skills (Gill et al., 2024; Haag et al., 2024). An element-based meta-analysis of adolescent mental-health interventions found a small overall effect on emotion regulation and identified several candidate components, although the component-level associations were not causal tests (Helland et al., 2022).

Drawing on both cognitive-developmental and social-domain theoretical traditions, prosocial moral development is conceptualized not merely as rule-based moral reasoning but as the integration of moral cognition, moral emotion, and tendencies toward moral action. Contemporary models increasingly emphasize that prosocial moral behavior during adolescence is scaffolded by affective processes, including empathic concern and compassion, rather than arising solely from abstract reasoning capacities. This affective-cognitive integration provides a theoretical bridge between compassion-based interventions and later moral-development outcomes (Cirimele et al., 2024; Qiu et al., 2024; Li et al., 2025a). Recent within-person evidence from Chinese adolescents further indicates that increases in moral disengagement can precede later reductions in prosocial behavior, although moral disengagement should not be treated as equivalent to emotion regulation (Wang et al., 2026).

### 2.2 Compassion and mindfulness interventions in youth

A substantial body of intervention research has examined mindfulness-based programs in school and community settings, generally reporting small-to-moderate improvements in attentional control, stress reactivity, and self-reported emotional well-being among adolescents. More recent large-scale evidence has been mixed: the MYRIAD trial found no superiority over usual school provision at one year; a three-arm trial found lower stress than an active relaxation control but not usual provision and no increase in self-kindness; and a multicomponent resilience program showed outcome-specific benefits at 24 weeks (Kuyken et al., 2022; Llistosella et al., 2024; Volanen et al., 2026). However, mindfulness-based curricula predominantly emphasize present-moment awareness and non-judgmental attention, with comparatively limited explicit content addressing interpersonal compassion or prosocial motivation (Kander et al., 2024; Shi and Cheung, 2024; Zhou and McLellan, 2024; Hayes et al., 2025; Juul et al., 2025).

Compassion-specific training programs, including cognitively based compassion training and compassion cultivation training, have been studied more frequently in adult populations. Findings suggest improvements in emotion regulation, reductions in self-criticism, and increases in prosocial behavior, including generosity in economic-game paradigms. Recent youth studies include a cluster-randomized compassion-based social–emotional learning trial with younger children, a small active-control trial of Mindful Self-Compassion for Teens, and an uncontrolled classroom pilot in early adolescents; collectively, these studies demonstrate feasibility while also underscoring limits in age generalizability, sample size, and causal inference (Seekis et al., 2023; Xavier et al., 2023; Bluth et al., 2024). Extensions of these protocols to children and adolescents remain comparatively nascent; existing studies are limited by small samples, single postintervention assessments, and reliance on passive or wait-list control conditions that cannot distinguish compassion-specific mechanisms from nonspecific effects of program participation, such as increased teacher attention, structured peer interaction, or expectancy effects. Furthermore, most existing trials have not used cluster-level randomization despite classroom-based delivery, raising concerns about contamination and inflated Type I error due to unmodeled nesting (Crompton et al., 2024; Maratos et al., 2024; Aguilera et al., 2025; Neuenschwander and von Gunten, 2025; Roberts et al., 2026).

The developmental suitability of compassion training for adolescents warrants particular theoretical attention. Because peer relationships and concerns about social status become especially salient during this period, compassion-based curricula may be particularly effective when they explicitly engage perspective-taking and frame experiences in terms of shared humanity. Such features may buffer against self-focused rumination and social comparison, both of which are prominent risk factors for emotion dysregulation during adolescence (Ward-Seidel et al., 2025). Peer context may also condition prosocial development: in a two-wave adolescent study, peer rejection predicted lower later prosocial behavior, while trait mindfulness buffered this association (Wang and Qi, 2023).

### 2.3 Emotion regulation and moral development linkage

Theoretical accounts of the relationship between emotion regulation and moral development have historically favored a unidirectional causal narrative in which regulatory capacity is treated as a prerequisite for sustained engagement in effortful, other-oriented moral reasoning and behavior. Evidence supporting this pathway indicates that emotion dysregulation, particularly difficulty in downregulating personal distress, is associated with empathic overarousal and subsequent withdrawal from prosocial engagement rather than approach-oriented helping behavior (Qiu et al., 2024; Ritgens et al., 2024; Carrizales et al., 2025; Li et al., 2025b).

An alternative, increasingly supported perspective proposes a reciprocal or bidirectional relationship in which engagement in prosocial moral behavior itself functions as an emotion-regulation strategy. Perspective-taking and compassionate engagement with others’ suffering may recruit reappraisal-like cognitive processes that redirect attention away from self-referential distress, thereby helping to regulate one’s own emotional state. This bidirectional formulation is consistent with broader theoretical models of mutually reinforcing developmental cascades between socioemotional competencies. Four-wave RI-CLPM evidence suggests that intentional self-regulation and prosocial behavior may be bidirectionally related in early adolescence, while three-wave evidence indicates that within-person elevations in empathy can precede later prosocial behavior (Memmott-Elison et al., 2020; Carrizales et al., 2021). Even so, directly comparable tests remain scarce, and differences in constructs, time intervals, and model specifications limit strong conclusions about reciprocity (Hamaker et al., 2015; van Meegen et al., 2024; Li et al., 2025b).

### 2.4 Conceptual model

Building on the theoretical and empirical foundations reviewed above, the present study proposes an integrated model in which compassion training exerts differential effects on the developmental trajectories of emotion regulation and prosocial moral development relative to a structurally matched active control, with these two developmental domains further hypothesized to exhibit reciprocal within-person influence over time. The following hypotheses guide the present investigation:

H1: Adolescents assigned to the compassion training condition will demonstrate significantly greater improvement in emotion regulation across T2, T3, and T4 relative to those in the active control condition, as reflected in a significant Group × Time interaction.

H2: Adolescents assigned to the compassion training condition will demonstrate significantly greater improvement in prosocial moral development across T2, T3, and T4 relative to those in the active control condition.

H3: The compassion training group will exhibit a piecewise growth pattern characterized by a steep positive slope during the intervention phase (T1–T2) followed by a shallower but sustained positive trajectory during the follow-up phase (T2–T3–T4), significantly differing from the trajectory observed in the active control group.

H4: At the within-person level, emotion regulation and prosocial moral development will show significant bidirectional cross-lagged prediction across adjacent time points, with the magnitude of these reciprocal effects hypothesized to be greater in the compassion training condition than in the active control condition.

Figure 1 summarizes the study’s four stated hypotheses within a single longitudinal framework. The H1 and H2 paths represent the expected condition-specific advantages of compassion training for emotion regulation and prosocial moral development, respectively. H3 distinguishes rapid change during the eight-week intervention (T1–T2) from the subsequent follow-up trajectory (T2–T4), whereas H4 specifies reciprocal within-person prediction across adjacent waves (ER_t → PMD_t + 1 and PMD_t → ER_t + 1). The conceptual model therefore separates intervention-related mean change from the temporal ordering of within-person fluctuations in the two developmental domains.

**Figure 1:**
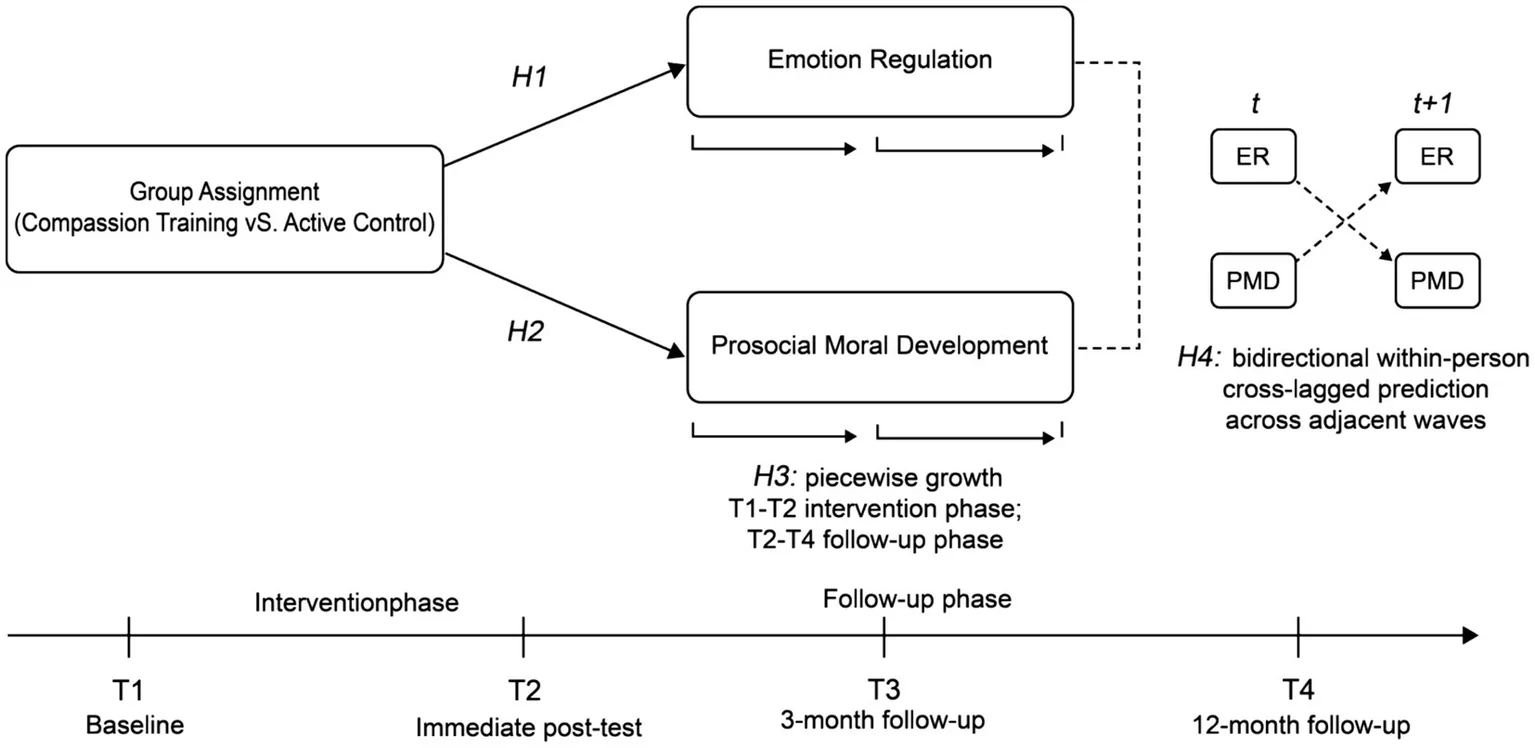
Conceptual model of hypothesized relations among group assignment, emotion regulation, and prosocial moral development.

## 3 Methods

### 3.1 Study design

This study employed a two-arm, parallel-group cluster-randomized controlled trial (cRCT) embedded in a four-wave longitudinal follow-up framework and conducted between February 2024 and June 2025. A cluster design was chosen over individual-level randomization because the intervention was delivered at the classroom level. Randomizing individual students within the same classroom would have created a high risk of contamination, as students receiving compassion training and those in the active control condition would inevitably interact, discuss course content, and potentially influence one another’s regulatory and prosocial responses. Classes therefore constituted the unit of randomization, whereas students remained the unit of analysis; repeated observations within students and students within classes were explicitly represented in the three-level outcome models (Campbell et al., 2012; Hopewell et al., 2025).

The longitudinal component comprised four assessment waves: a preintervention baseline (T1), an immediate postintervention assessment (T2) at the conclusion of the eight-week program, a short-term follow-up (T3) three months after intervention completion, and a long-term follow-up (T4) twelve months after intervention completion. This four-wave structure served three distinct inferential purposes rather than a single omnibus comparison. First, the T1–T2 interval isolated the immediate intervention effect under conditions of maximal program exposure. Second, the T2–T3 interval evaluated the durability of any observed effect after direct program contact had ceased, addressing the common criticism that psychosocial intervention effects dissipate rapidly following termination. Third, the T3–T4 interval extended this assessment of durability across a developmentally meaningful period, allowing the study to distinguish skill consolidation from transient postintervention enthusiasm. Embedding this temporal logic in the design allowed the piecewise growth modeling strategy to map explicitly onto the intervention and follow-up phases rather than assuming a single linear trajectory across the entire study period.

As illustrated in Figure 2, the four assessment waves are anchored to the intervention period rather than to fixed calendar time, ensuring that the T2, T3, and T4 intervals are equivalent in duration across both study arms despite any minor scheduling variation across the four participating schools. This anchoring strategy was essential given the cluster design, as intervention start dates could not be perfectly synchronized across 34 classes distributed across four institutions.

**Figure 2:**
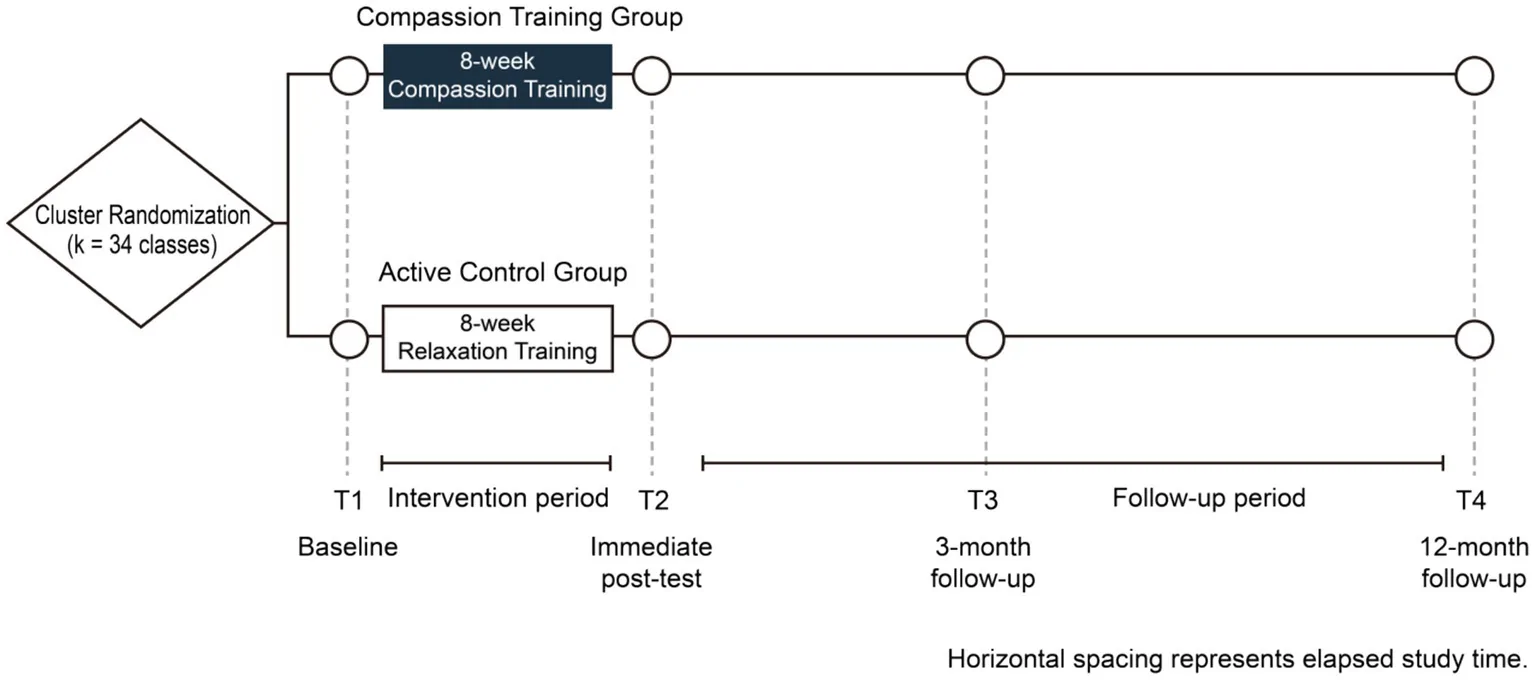
Study design and assessment timeline (T1–T4).

Figure 2 depicts the randomization of 34 classroom clusters into two duration- and contact-matched conditions. Both study arms were assessed at the same four waves, with the intervention ending at T2. The deliberately unequal horizontal spacing represents elapsed study time: eight weeks from T1 to T2, three months from T2 to T3, and a further nine months from T3 to T4. This temporal alignment permits immediate intervention effects to be distinguished from short- and long-term maintenance.

### 3.2 Participants and setting

Participants were recruited from four secondary schools in China, selected to represent variation in institutional resources and regional characteristics while remaining comparable in curricular structure. Students aged 12 to 18 years enrolled in participating classes were considered for participation, subject to the exclusion criteria below. Exclusion criteria comprised current clinical treatment for a diagnosed psychiatric condition, exposure to a major traumatic event within the preceding six months, and inability to reliably complete self-report assessments across the four planned waves. Recruitment yielded 876 students nested within 34 classes, consistent with the planning target range of 800 to 1,000 students and 30 to 40 clusters established during power analysis planning.

The program was designed as a universal school-based developmental intervention, with research eligibility screening, rather than as treatment for psychiatric disorders or trauma. The clinical-treatment and trauma exclusions were intended to reduce potential distress during repeated emotion-focused and compassion exercises and to avoid interference with an ongoing individualized treatment plan. Major traumatic events included the death of an immediate family member, a serious accident or physical injury, violence, exposure to a severe natural disaster, or another major event judged by a school mental-health teacher or guardian to be associated with substantial ongoing distress. Screening used a parent or guardian form and a brief student self-report, with verification by a school mental-health teacher when needed. Students were not asked to recount traumatic experiences in detail. The research team did not make new psychiatric diagnoses. Current clinical treatment was identified from an existing professional diagnosis and treatment status.

Students meeting these exclusions were not enrolled in research assessments or the analyzed trial cohort. Classes, rather than individual students, remained the units of randomization. Research exclusion did not remove access to routine school education or psychological support. Where activities formed part of normal school provision, schools could determine appropriate participation. If a school mental-health teacher considered a particular exercise unsuitable, an alternative activity or referral could be arranged. No data from these excluded students entered the research database.

Of 950 students assessed for eligibility, 74 were excluded and 876 were randomized across 34 classes: 447 students in 17 compassion-training classes and 429 in 17 active-control classes (Figure 3). At T2, 423 and 419 students completed assessment; at T3, 395 and 386; and at T4, 270 and 252, respectively. Overall retention was 96.1%, 89.2%, and 59.6% at T2, T3, and T4. Cumulative T4 losses were 177 students per condition, corresponding to 39.6 and 41.3% attrition. The ITT population comprised all 876 randomized students, including 522 four-wave completers. Table 1 summarizes assessment completion and cumulative missing assessments.

**Figure 3:**
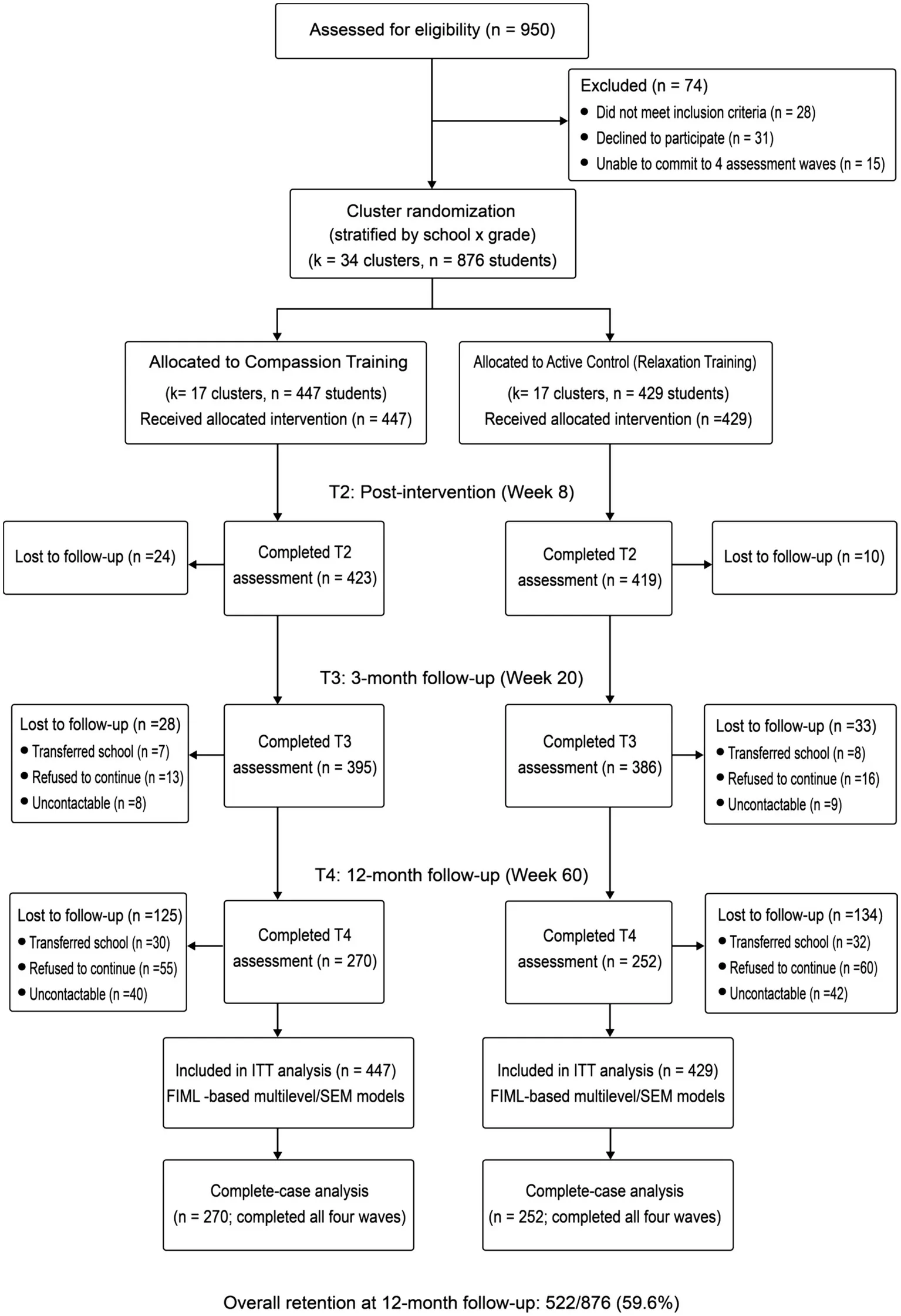
CONSORT flow diagram of participant enrollment and follow-up.

**Table 1:** Assessment completion and cumulative missing assessments by condition.

Wave	Compassion training Completed/missing	Active control Completed/missing	Total completed *n* (%)
T1	447/0	429/0	876 (100.0)
T2	423/24	419/10	842 (96.1)
T3	395/52	386/43	781 (89.2)
T4	270/177	252/177	522 (59.6)

Missing = randomized − completed at each wave. Counts refer to assessment completion. Reasons for loss to follow-up included transfer, refusal to continue, and inability to contact.

### 3.3 Randomization and allocation concealment

Randomization was conducted at the class level using a computer-generated sequence and was stratified by school and grade to balance institutional context and developmental stage, both of which were expected to influence baseline emotion regulation and moral reasoning capacity. Within each school-by-grade stratum, classes were allocated in a 1:1 ratio to the compassion training condition or the active control condition. An independent statistician generated and implemented the allocation sequence and had no role in recruitment, teaching, or assessment. Allocation was concealed from classroom teachers until immediately before the program began, thereby minimizing selection bias at enrollment (Campbell et al., 2012).

### 3.4 Sample size and power analysis

Sample-size planning considered an anticipated effect of d = 0.35–0.45, an ICC of 0.02–0.05, 30–40 classes, an average class size of 22–25 students, and 25–35% attrition (Table 2). With equal cluster sizes, the design effect is 1 + (m − 1)ICC; these ranges imply design effects of approximately 1.42–2.20. A Monte Carlo planning simulation used 5,000 replications, α = 0.05, ICC = 0.03, 34 clusters, a mean cluster size of 25.8, and an anticipated effect of d = 0.40. Estimated power was approximately 80% or higher under these assumptions. The achieved mean class size was 25.76 students. Power depended on the number and distribution of classes as well as individual sample size.

**Table 2:** Parameters and assumptions for sample size and power estimation.

Parameter	Value/Assumption
Anticipated effect size (Cohen’s d)	Moderate (d ≈ 0.35–0.45)
Intraclass correlation coefficient (ICC)	0.02–0.05
Target number of clusters (classes)	30–40
Average cluster size	22–25 students
Initial recruitment target	800–1,000 students
Actual recruitment	876 students
Anticipated attrition rate	25–35%
Target statistical power	≥ 0.80
Power evaluation method	cRCT design-effect adjustment; Monte Carlo simulation for RI-CLPM and LGCM

At T4, 522 of 876 students remained, yielding 59.6% retention and 40.4% attrition, which exceeded the 25–35% planning assumption. The 34 randomized classes determined the number of independent treatment units. ITT analyses included incomplete records under the relevant missing-data assumptions, but the additional attrition reduced follow-up information relative to the planning scenario.

### 3.5 Intervention conditions

#### 3.5.1 Compassion training group

Students assigned to the compassion-training condition received eight weekly 45-min classroom sessions over eight consecutive weeks, supplemented by structured home practice. The curriculum integrated CFT/CMT principles, self-compassion, social–emotional learning, and perspective-taking, as described in Section 2.1. It progressed from self-compassion and non-judgmental self-awareness to compassionate attention toward familiar others and then toward unfamiliar others through a shared-humanity framework. Sessions integrated guided experiential exercises, emotion-labeling tasks, perspective-taking, and role-play linking compassion to prosocial decision-making. Table 3 details the weekly content.

**Table 3:** Weekly compassion-training and active-control curriculum.

Week	Compassion training, activities, and home practice	Active control
1	Emotional awareness and introduction to compassion. Activities: Check-in, emotion identification, guided awareness, discussion, and assignment of home practice. Home practice: Record an emotional experience and its bodily responses.	Diaphragmatic breathing
2	Self-compassion and non-judgment. Activities: Review, identification of self-criticism, compassionate self-talk, and practice. Home practice: Practice compassionate self-talk for 10–15 min.	Progressive muscle relaxation
3	Emotion identification and labeling. Activities: Emotion vocabulary, intensity ratings, bodily cues, and emotion naming. Home practice: Keep an emotion-labeling diary.	Breathing and body relaxation
4	Perspective-taking. Activities: Scenario stories, role reversal, inference about others’ viewpoints, and group discussion. Home practice: Describe a conflict from two perspectives.	Guided imagery
5	Compassion toward familiar others. Activities: Recognition of others’ difficulties, compassionate imagery, and role-play of supportive responses. Home practice: Carry out one supportive action each day.	Progressive relaxation
6	Compassion toward unfamiliar others. Activities: Scenarios involving unfamiliar people, discussion of common needs, and compassionate attention. Home practice: Record a need noticed in an unfamiliar person.	Guided imagery
7	Shared humanity and prosocial decision-making. Activities: Shared humanity, moral dilemmas, consideration of costs and needs, and role-play. Home practice: Carry out a planned prosocial action.	Combined relaxation practice
8	Integration and everyday application. Activities: Review of skills, integrated practice, a personal action plan, and maintenance strategies. Home practice: Develop a plan for continued practice.	Review and independent relaxation

Each condition comprised eight weekly 45-min sessions with 10–15 min of prescribed daily home practice. The compassion activities and home assignments are listed by session. Active-control home practice used relaxation exercises from the control curriculum, which did not explicitly teach compassion, perspective-taking, or moral reasoning.

#### 3.5.2 Active control group

Students in the active-control condition received eight weekly 45-min relaxation-training sessions with 10–15 min of daily home practice. Contact frequency, session duration, and prescribed home-practice duration were planned to match the compassion-training condition (Table 4). Activities comprised diaphragmatic breathing, progressive muscle relaxation, guided imagery, and combined relaxation practice, with weekly content specified in Table 3. The curriculum did not explicitly teach compassion, perspective-taking, or moral reasoning. Relaxation can itself affect emotional experience; the comparison therefore estimates the incremental effect of compassion training relative to a relaxation program.

**Table 4:** Comparison of intervention and active control conditions on structural parameters.

Parameter	Compassion training	Active control
Duration	8 weeks	8 weeks
Session frequency	1 session/week	1 session/week
Teacher-student contact time	45 min/session (planned matching)	45 min/session (planned matching)
Home practice requirement	10–15 min/day (prescribed)	10–15 min/day (prescribed)
Core content	Compassion, perspective-taking, moral reasoning	Breathing, progressive muscle relaxation, imagery
Explicit reappraisal/compassion instruction	Explicit	No explicit compassion curriculum; relaxation may regulate emotion

Table 4 summarizes the planned structural matching and Table 3 presents the weekly curriculum. Matching reduces concern about unequal contact or prescribed dosage but does not demonstrate equivalence in expectancy, facilitator allegiance, or achieved home-practice adherence.

#### 3.5.3 Facilitators, training, and home practice

A total of four facilitators delivered the two conditions according to a prespecified classroom schedule. They comprised two school mental-health teachers, one educational psychology researcher, and one education teacher with experience in school-based social–emotional learning. All had at least two years of experience teaching adolescents or providing psychoeducation. All four completed standardized training in both curricula.

The 12-h training comprised two 6-h modules. The first covered the study protocol and safeguarding (1 h), compassion and CFT theory (1.5 h), the eight-session curriculum (1.5 h), demonstration of guided exercises (1 h), and management of classroom risks (1 h). The second covered the active-control protocol (1.5 h), role-play and teaching rehearsal (2 h), the fidelity checklist (1 h), home-practice management (0.5 h), and standardized assessment with feedback (1 h). During the intervention, the project lead and a psychology professional with experience in adolescent interventions jointly provided weekly supervision. There were eight supervision sessions of approximately 60 min each, totaling approximately 8 h.

Both conditions prescribed 10–15 min of daily home practice and collected weekly practice logs. The compassion condition had a log return rate of 88.6%, with reported practice averaging 5.1 days per week and 12.4 min per day. Corresponding values in the active-control condition were 87.9%, 5.0 days per week, and 12.1 min per day. The logs were used for implementation monitoring. Individual practice dose was not included in the primary outcome models.

### 3.6 Intervention fidelity

Approximately 25% of intervention sessions were randomly selected for fidelity assessment. Sessions from both conditions were recorded and evaluated by two independent raters who were not involved in intervention delivery. A standardized 5-point checklist assessed content adherence, facilitator competence, and student engagement. Inter-rater reliability was estimated using a two-way random-effects, average-measures intraclass correlation coefficient, ICC(2,k), with k = 2 raters. Raters were blinded to the study hypotheses but could not be blinded to intervention content.

### 3.7 Measures

#### 3.7.1 Emotion regulation

Emotion regulation (ER) was assessed using the 10-item Emotion Regulation Questionnaire (ERQ; Gross and John, 2003), which covers cognitive reappraisal and expressive suppression. Cognitive Reappraisal (CR) comprises items 1, 3, 5, 7, 8, and 10, and Expressive Suppression (ES) comprises items 2, 4, 6, and 9. Responses are scored from 1 to 7. No items are reverse-scored. Higher subscale scores indicate more frequent use of the corresponding strategy.

The original ERQ uses agreement anchors from strongly disagree to strongly agree. An English-language example of expressive suppression is “I control my emotions by not expressing them” (Gross and John, 2003). In a study of 639 Chinese adolescents aged 12–18, Sai et al. (2016) reported internal consistency of 0.72 for reappraisal and 0.70 for suppression. Reappraisal was negatively associated with depressive symptoms, whereas suppression was not significantly associated with them.

Internal consistency for CR at T1, T2, T3, and T4 was α = 0.84, 0.86, 0.85, and 0.87 and ω = 0.85, 0.87, 0.86, and 0.88, respectively. Corresponding estimates for ES were α = 0.78, 0.80, 0.79, and 0.81 and ω = 0.79, 0.81, 0.80, and 0.82.

#### 3.7.2 Prosocial moral development

Prosocial moral development (PMD) was a study-specific outcome comprising prosocial behavioral tendencies, moral reasoning, and moral identity. It encompassed behavioral, cognitive, and identity-related aspects of moral functioning. Prosocial behavioral tendencies were assessed with six items using a five-point response scale. Moral reasoning was assessed with six scenarios, with reasons for action scored from 1 to 5 along a progression from self-interest to concern for needs or values. Moral identity was assessed with eight items using a five-point Likert response scale.

For prosocial behavior, internal consistency at T1, T2, T3, and T4 was α = 0.82, 0.84, 0.85, and 0.84 and ω = 0.83, 0.85, 0.86, and 0.85, respectively. For moral reasoning, the corresponding values were α = 0.76, 0.79, 0.78, and 0.80 and ω = 0.77, 0.80, 0.79, and 0.81. For moral identity, they were α = 0.85, 0.87, 0.86, and 0.88 and ω = 0.86, 0.88, 0.87, and 0.89.

#### 3.7.3 Baseline characteristics and covariates

Baseline characteristics included age, gender, socioeconomic status, empathic concern, and social desirability. Age, gender, and socioeconomic status were included as covariates in the three-level outcome models. Baseline empathic concern and social desirability were additionally included in the imputation model. At T1, internal consistency was α = 0.83 and ω = 0.84 for empathic concern and α = 0.75 and ω = 0.76 for social desirability. These auxiliary variables were measured at baseline only.

### 3.8 Missing data strategy

The primary analyses followed the intention-to-treat principle. FIML was the primary missing-data approach for models estimated within the structural-equation framework. For analyses requiring multiply imputed datasets, 50 imputations were generated. The imputation model included treatment condition, assessment wave, ER and PMD scores at all available waves, age, gender, socioeconomic status, baseline empathic concern, and social desirability. Estimates were pooled using Rubin’s rules. FIML and the primary imputation analyses assumed that missingness was conditionally independent of unobserved outcomes given observed information (MAR; Enders, 2022). Delta-adjusted pattern-mixture sensitivity analyses examined departures from MAR using δ = −0.25, −0.50, and −0.75 SD. These scenarios represented progressively lower unobserved outcomes and assessed the sensitivity of the Group × T4 estimates.

### 3.9 Analytic strategy

The analyses addressed three complementary questions: differential average change, phase-specific growth, and within-person temporal association. They do not provide interchangeable tests of intervention mechanisms.

#### 3.9.1 Hierarchical linear modeling

Three-level hierarchical models represented repeated assessments within students and students within classes. Active control and T1 were the reference categories. Each Group × Time coefficient estimated the difference between conditions in change from baseline to that wave. The between-condition difference in outcome levels at a given wave was the group main effect plus the corresponding interaction. Differential change was standardized using the pooled baseline SD: SD = sqrt[((447–1)SD_CT^2^ + (429–1)SD_AC^2^)/(447 + 429–2)]. Approximate 95% confidence intervals were calculated as b ± 1.96 × SE; standardized interval limits were divided by the pooled baseline SD. These normal-approximation intervals treated the estimated baseline SD as fixed and did not incorporate a finite-cluster degrees-of-freedom correction.

#### 3.9.2 Latent growth curve modeling

Piecewise latent growth curve models decomposed trajectories into an intervention-phase slope (T1–T2) and a follow-up-phase slope (T2–T3–T4), testing H3. The intervention-phase factor was coded 0, 1, 1, and 1 at T1–T4, respectively. The follow-up factor was coded 0, 0, 0.25, and 1.00, reflecting relative elapsed time from immediate post-test to the 3- and 12-month follow-ups. The intervention slope therefore represented change over the eight-week program, and the follow-up slope represented change over the 12-month follow-up period.

#### 3.9.3 Measurement invariance testing

Configural, metric, and scalar invariance models tested measurement equivalence jointly across the four waves and both conditions. Invariance was evaluated using a CFI decrease no larger than 0.010 and an RMSEA increase no larger than 0.015 between successive models. Absolute fit indices and sequential changes are presented in Table 5. These tests assessed the comparability of the constructs across waves and conditions (Little, 2024).

**Table 5:** Fit indices for measurement invariance models across time and group.

Construct	Model	χ^2^	df	CFI	TLI	RMSEA	SRMR	ΔCFI	ΔRMSEA
Emotion regulation	Configural	386.42	224	0.972	0.964	0.037	0.034	—	—
	Metric	402.17	242	0.97	0.965	0.036	0.036	−0.002	−0.001
	Scalar	428.55	260	0.966	0.963	0.036	0.038	−0.004	0.000
Prosocial moral development	Configural	411.83	236	0.968	0.96	0.039	0.035	—	—
	Metric	430.26	255	0.965	0.961	0.038	0.037	−0.003	−0.001
	Scalar	459.94	274	0.961	0.958	0.038	0.04	−0.004	0.000

#### 3.9.4 Random intercept cross-lagged panel model

An RI-CLPM separated stable between-person differences from within-person deviations to examine H4 (Hamaker et al., 2015). Autoregressive paths represented within-domain temporal stability, and cross-lagged paths represented prediction between ER and PMD across successive assessments. The multigroup analysis estimated condition-specific cross-lagged coefficients, standard errors, 95% confidence intervals, and *p* values. Wald tests compared corresponding cross-lagged parameters between conditions. Because assessment intervals differed in duration, coefficients were interpreted within each interval rather than as directly comparable rates of developmental change.

Figure 4 formalizes the decomposition required for the RI-CLPM. Each observed ER or PMD score is represented separately from its time-specific within-person latent deviation, while RI-ER and RI-PMD capture stable between-person differences across the four waves. Their double-headed covariance represents the trait-level association between the two constructs. Autoregressive paths estimate temporal carry-over within each domain, cross-lagged paths estimate whether deviations in one domain predict subsequent deviations in the other, and within-wave residual covariances account for concurrent coupling. In the multi-group model, equality-constrained parameter comparisons, rather than differences in within-group significance, are required to determine whether these pathways differ between conditions (Mulder and Hamaker, 2021).

**Figure 4:**
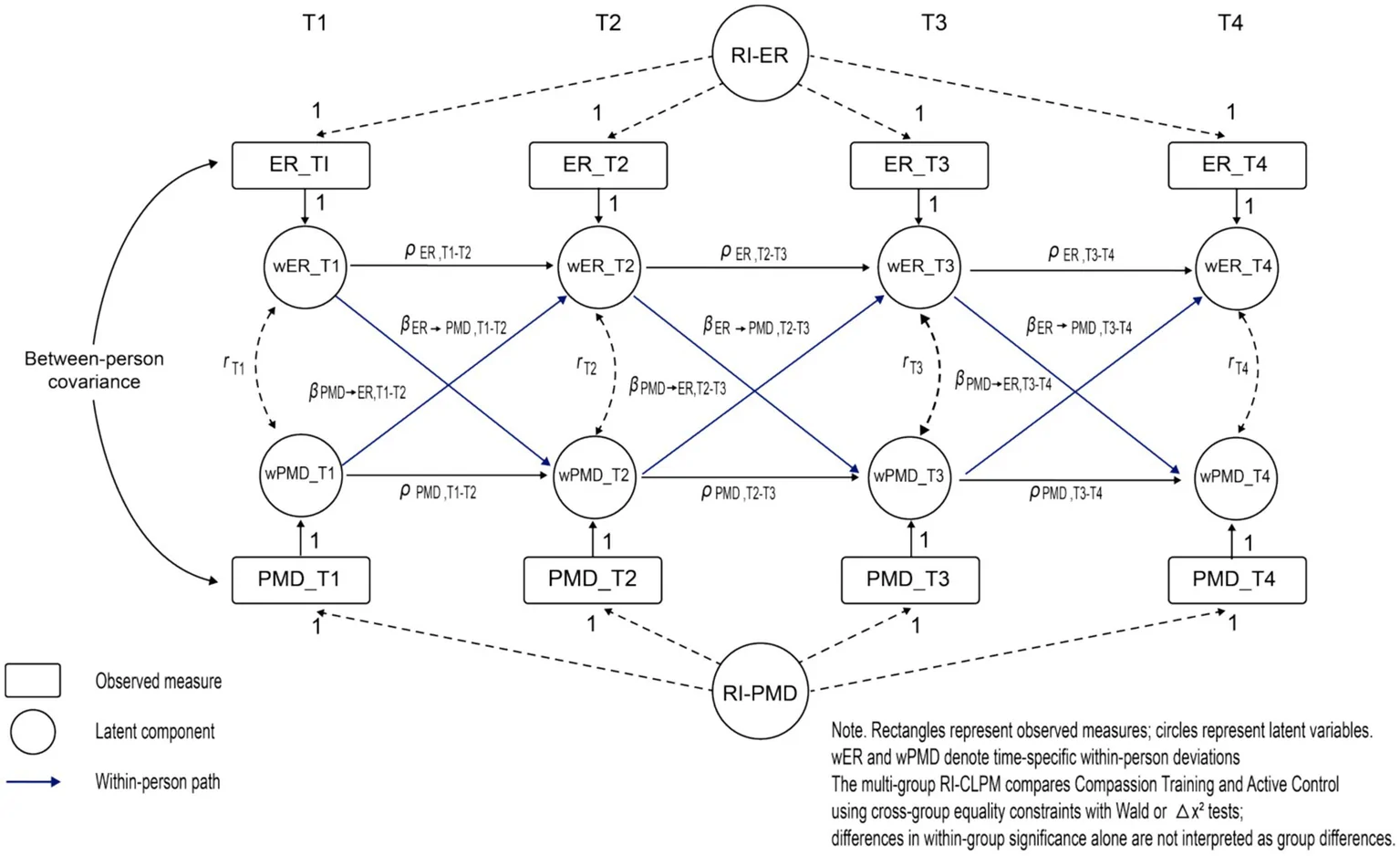
Hypothesized RI-CLPM path diagram.

Structural equation models, including the growth, measurement-invariance, and RI-CLPM analyses, were estimated in Mplus version 8.11 using robust maximum likelihood (MLR). Classroom dependence was accommodated using TYPE = COMPLEX with class as the clustering variable, providing cluster-adjusted standard errors and model test statistics. Missing outcomes were handled using FIML under MAR. This specification accounts for classroom clustering, but does not itself establish small-sample accuracy with 34 classes. Finite-cluster sensitivity and school-context effects remain relevant; four schools alone provide limited information for estimating population-level school variation.

### 3.10 Ethics statement

The study protocol was approved by the institutional ethics committee at Nanjing Normal University (approval no. 22JJD880031). For participants who were minors, written informed consent was obtained from their parents or legal guardians, and the participating students provided assent. Participants assigned to the active-control condition were offered access to the compassion-training curriculum after completion of the T4 assessment.

## 4 Results

### 4.1 Baseline equivalence

The conditions had similar baseline characteristics (Table 6). None of the baseline comparisons was statistically significant. These comparisons describe the sample at allocation and were not used as tests of equivalence.

**Table 6:** Baseline characteristics by group.

Variable	Compassion training (*n* = 447)	Active control (*n* = 429)	Test statistic	*p*
Age, years, M (SD)	14.62 (1.78)	14.55 (1.81)	t = 0.57	0.571
Female, %	51.9	49.7	χ^2^ = 0.42	0.517
Socioeconomic status (z-score), M (SD)	0.03 (0.98)	−0.02 (1.01)	t = 0.72	0.474
Baseline emotion regulation, M (SD)	32.14 (6.87)	31.89 (7.02)	t = 0.53	0.598
Baseline prosocial moral development, M (SD)	64.37 (9.45)	63.98 (9.61)	t = 0.59	0.556
Baseline empathic concern, M (SD)	27.66 (5.23)	27.41 (5.38)	t = 0.68	0.499
Social desirability, M (SD)	15.82 (4.11)	16.05 (4.24)	t = −0.80	0.426

Subsequent outcome analyses accounted for baseline-to-wave change and classroom clustering.

### 4.2 Attrition analysis

Table 7 compares students who completed all four assessment waves (*n* = 522) with those who did not (*n* = 354) on baseline characteristics, in order to evaluate whether attrition occurred systematically with respect to demographic factors, treatment condition, or baseline outcome levels.

**Table 7:** Comparison of four-wave completers and non-completers on baseline characteristics.

Variable	Completers (*n* = 522)	Non-completers (*n* = 354)	Test statistic	*p*
Age, years, M (SD)	14.71 (1.74)	14.39 (1.86)	t = 2.51	0.012
Female, %	54.2	46.3	χ^2^ = 5.68	0.017
Socioeconomic status (z-score), M (SD)	0.09 (0.95)	−0.14 (1.04)	t = 3.44	< 0.001
Baseline emotion regulation, M (SD)	32.21 (6.79)	31.68 (7.11)	t = 1.11	0.267
Baseline prosocial moral development, M (SD)	64.52 (9.33)	63.71 (9.77)	t = 1.24	0.216
Compassion training condition, %	51.7	50.0	χ^2^ = 0.25	0.616

Four-wave completers were older, more often female, and had higher socioeconomic status than non-completers (Table 7). Baseline outcome scores and treatment allocation did not differ significantly between these groups. These patterns indicated selection on measured characteristics. The missing-data analyses included these characteristics, and delta-adjusted sensitivity analyses examined potential dependence on unobserved outcomes.

### 4.3 Intervention Fidelity results

Fidelity ratings were similar between conditions in the sampled sessions (Table 8). Overall inter-rater reliability for the average of two raters was ICC (2,2) = 0.86 (95% CI 0.79–0.91). Between-condition tests did not detect differences in fidelity ratings. These findings suggest comparable implementation in the sampled sessions, while the sampling design limits conclusions about all delivered sessions and individual home practice.

**Table 8:** Fidelity ratings by group.

Fidelity dimension (1–5 scale)	Compassion Training, M (SD)	Active Control, M (SD)	t	*p*
Content adherence	4.52 (0.34)	4.47 (0.39)	0.83	0.41
Facilitator competence	4.38 (0.41)	4.41 (0.37)	−0.44	0.663
Student engagement quality	4.21 (0.48)	4.15 (0.52)	0.71	0.481
Overall fidelity score	4.37 (0.33)	4.34 (0.36)	0.51	0.611

### 4.4 Overall intervention effects

Positive Group × Time terms at T2, T3, and T4 indicated greater change from T1 under compassion training, supporting H1 and H2 (all *p* < 0.001; Table 9). For ER, differential change was 3.417 points at T2 and 2.203 at T4; for PMD, it was 4.216 and 2.649, respectively. Baseline-SD-standardized differential changes were 0.492, 0.413, and 0.317 for ER, and 0.442, 0.375, and 0.278 for PMD at T2, T3, and T4 (Table 10). Thus, the relative advantages were smaller at later assessments but remained positive. The class ICCs were 0.027 for ER and 0.029 for PMD.

**Table 9:** Three-level model estimates and approximate 95% confidence intervals.

Predictor	Emotion Regulation b (SE) [approx. 95% CI]	*p*	Prosocial Moral Development b (SE) [approx. 95% CI]	*p*
Intercept	31.940 (0.412) [31.132, 32.748]	< 0.001	63.870 (0.478) [62.933, 64.807]	< 0.001
Group (compassion training)	0.187 (0.583) [−0.956, 1.330]	0.749	0.312 (0.612) [−0.888, 1.512]	0.61
Time T2 (vs. T1)	1.204 (0.298) [0.620, 1.788]	< 0.001	1.532 (0.367) [0.813, 2.251]	< 0.001
Time T3 (vs. T1)	0.876 (0.312) [0.264, 1.488]	0.005	1.098 (0.389) [0.336, 1.860]	0.005
Time T4 (vs. T1)	0.593 (0.334) [−0.062, 1.248]	0.076	0.734 (0.415) [−0.079, 1.547]	0.077
Group × T2	3.417 (0.421) [2.592, 4.242]	< 0.001	4.216 (0.512) [3.212, 5.220]	< 0.001
Group × T3	2.865 (0.446) [1.991, 3.739]	< 0.001	3.578 (0.541) [2.518, 4.638]	< 0.001
Group × T4	2.203 (0.478) [1.266, 3.140]	< 0.001	2.649 (0.573) [1.526, 3.772]	< 0.001
Age	0.142 (0.089) [−0.032, 0.316]	0.111	0.187 (0.102) [−0.013, 0.387]	0.068
Gender (female)	0.891 (0.267) [0.368, 1.414]	< 0.001	1.124 (0.312) [0.512, 1.736]	< 0.001
Socioeconomic status	0.318 (0.134) [0.055, 0.581]	0.018	0.276 (0.156) [−0.030, 0.582]	0.077
Class-level variance	0.68	—	0.94	—
Student-level variance	14.85	—	19.87	—
Residual (occasion) variance	9.28	—	11.36	—
Intraclass correlation (class)	0.027	—	0.029	—

Approximate 95% CIs = b ± 1.96 × SE. These normal-approximation intervals do not incorporate a finite-cluster degrees-of-freedom correction.

**Table 10:** Derived between-condition level differences and standardized differential change.

Outcome	Wave	Adjusted level difference	Standardized differential change [approx. 95% CI]
ER	T2	3.604	0.492 [0.373, 0.611]
ER	T3	3.052	0.413 [0.287, 0.538]
ER	T4	2.390	0.317 [0.182, 0.452]
PMD	T2	4.528	0.442 [0.337, 0.548]
PMD	T3	3.890	0.375 [0.264, 0.487]
PMD	T4	2.961	0.278 [0.160, 0.396]

Positive values favor compassion training. Level difference = group main effect + Group × Time term. Standardized differential change = interaction/pooled baseline SD (ER 6.944; PMD 9.529). Interval limits condition on the baseline SD estimate. Level differences and differential changes are distinct estimands.

### 4.5 Growth trajectories

Figure 5A shows the piecewise trajectory for emotion regulation. The compassion training group exhibited a pronounced intervention-phase increase (Slope 1 = 3.52), compared with only a small positive change in the active control group (Slope 1 = 0.184); the between-group slope difference was significant (*p* < 0.001). During follow-up, both trajectories declined, with a more negative slope in the compassion group (−0.318 vs. −0.089; *p* = 0.032). This pattern indicates partial attenuation after the immediate post-test peak, although the compassion group remained above its baseline level and the active-control trajectory at T4.

**Figure 5:**
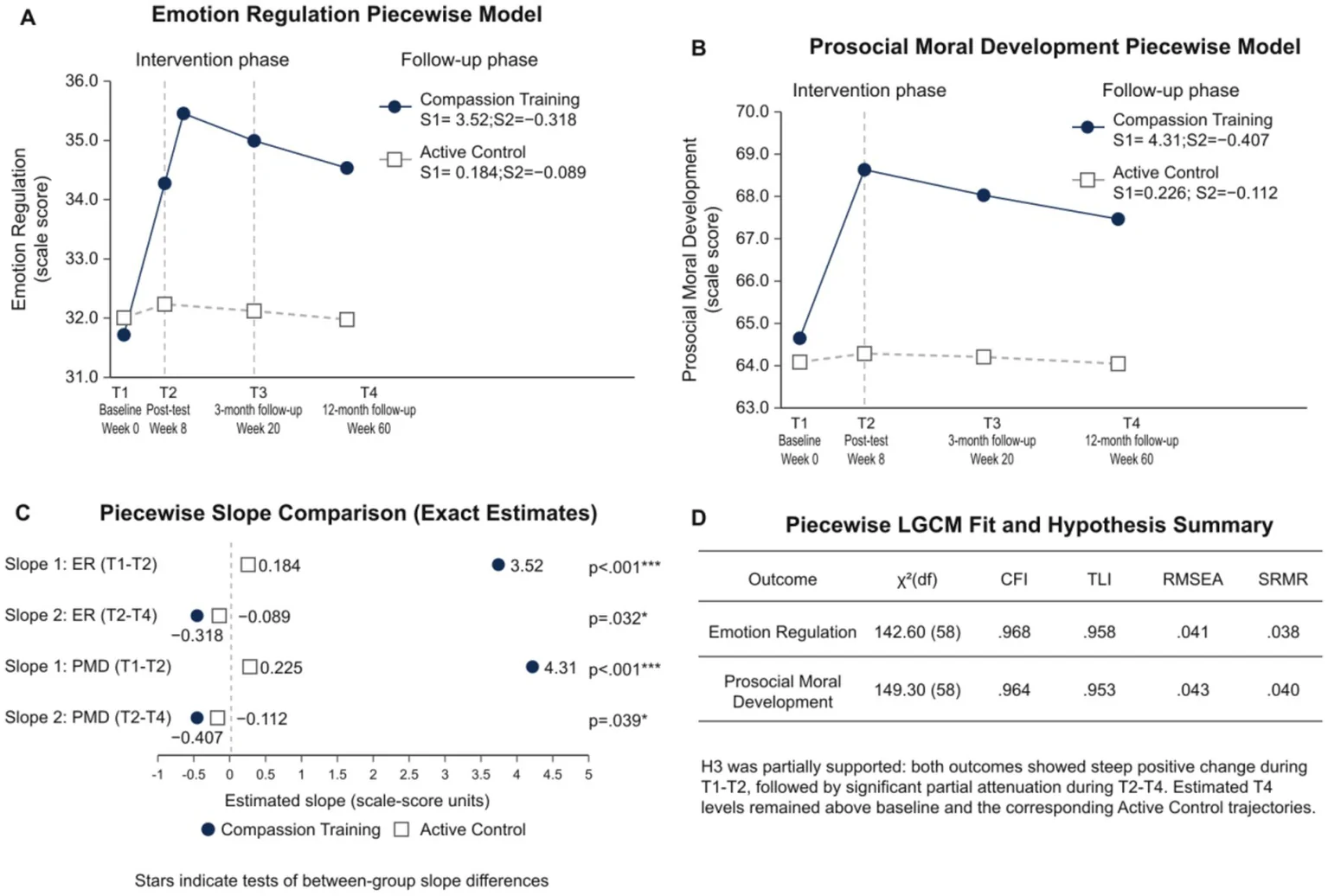
Piecewise growth trajectories and model estimates for emotion regulation and prosocial moral development. **(A)** Emotion regulation. **(B)** Prosocial moral development. **(C)** Between-condition slope comparisons. **(D)** Model fit and hypothesis summary. H3 was partially supported: intervention-period gains were followed by attenuation during follow-up.

Figure 5B demonstrates the corresponding pattern for prosocial moral development. The intervention-phase increase was markedly larger under compassion training than active control (4.31 vs. 0.226; *p* < 0.001). Follow-up slopes were negative in both groups and more negative in the compassion condition (−0.407 vs. −0.112; *p* = 0.039), indicating partial loss of the immediate gain rather than continued positive growth. Nevertheless, the estimated T4 level remained above baseline and above the active-control trajectory.

Figure 5C compares the four piecewise slope parameters. Both intervention-phase between-condition contrasts were significant (*p* < 0.001). The follow-up-phase contrasts were also significant for ER (*p* = 0.032) and PMD (*p* = 0.039), reflecting steeper post-intervention decline in the compassion condition.

Figure 5D reports acceptable fit for both piecewise models: emotion regulation, χ^2^(58) = 142.60, CFI = 0.968, TLI = 0.958, RMSEA = 0.041, and SRMR = 0.038; prosocial moral development, χ^2^(58) = 149.30, CFI = 0.964, TLI = 0.953, RMSEA = 0.043, and SRMR = 0.040. Taken together, Panels A–D indicate that only the intervention-period component of H3 was supported; the prediction of sustained positive follow-up growth was not supported. Negative follow-up slopes, especially in the compassion group, describe attenuation rather than continued beneficial growth.

Table 11 summarizes the piecewise growth parameters. Intervention-period slope contrasts favored compassion training for ER and PMD (both *p* < 0.001). Follow-up slopes were negative and more negative under compassion training (ER: −0.318 vs. −0.089, *p* = 0.032; PMD: −0.407 vs. −0.112, *p* = 0.039). These results supported the intervention-period component of H3 but not its prediction of continued positive growth during follow-up.

**Table 11:** Latent growth parameters for intervention and follow-up phases.

Outcome	Parameter	Compassion training	Active control	z (Δ)	*p*
Emotion regulation	Intercept mean	31.87	32.05	−0.41	0.682
	Intercept variance	13.42	14.05	—	—
	Slope 1 (T1 → T2) mean	3.52	0.184	9.87	< 0.001
	Slope 1 variance	2.87	1.02	—	—
	Slope 2 (T2 → T4) mean	−0.318	−0.089	−2.14	0.032
	Slope 2 variance	1.45	0.78	—	—
Prosocial moral development	Intercept mean	64.29	63.91	0.37	0.711
	Intercept variance	21.36	22.08	—	—
	Slope 1 (T1 → T2) mean	4.31	0.226	10.42	< 0.001
	Slope 1 variance	3.64	1.31	—	—
	Slope 2 (T2 → T4) mean	−0.407	−0.112	−2.06	0.039
	Slope 2 variance	1.98	0.94	—	—

### 4.6 Measurement invariance results

The invariance models showed small changes in fit across successive constraints (Table 5). For ER, ΔCFI values were −0.002 and −0.004, ΔRMSEA values were −0.001 and 0.000, and ΔSRMR values were +0.002 and +0.002. For PMD, the corresponding changes were −0.003 and −0.004, −0.001 and 0.000, and +0.002 and +0.003. The CFI and RMSEA changes met the specified criteria, supporting measurement equivalence across waves and conditions under the fitted models.

### 4.7 Within-person cross-lagged relations

The RI-CLPM indicated a consistent ER-to-PMD temporal association in the compassion-training condition (Table 12). Within-person elevations in ER predicted higher subsequent PMD from T1 to T2 (β = 0.220, SE = 0.041, 95% CI [0.140, 0.300], *p* < 0.001), T2 to T3 (β = 0.190, SE = 0.041, 95% CI [0.110, 0.270], *p* < 0.001), and T3 to T4 (β = 0.150, SE = 0.051, 95% CI [0.050, 0.250], *p* = 0.003). The reverse PMD-to-ER paths were significant from T1 to T2 (β = 0.110, *p* = 0.017) and T2 to T3 (β = 0.090, *p* = 0.028), but not from T3 to T4 (β = 0.080, *p* = 0.117). In the active-control condition, the T1-to-T2 ER-to-PMD path had a rounded *p* value of 0.050; the other five paths had *p* values above 0.05.

**Table 12:** Condition-specific RI-CLPM cross-lagged estimates and between-condition Wald tests.

Interval/path	Condition	β	SE	95% CI	*p*	Wald p
T1 → T2 ER → PMD	Compassion	0.220	0.041	0.140–0.300	<0.001	0.067
	Control	0.100	0.051	0.000–0.200	0.050	
T1 → T2 PMD → ER	Compassion	0.110	0.046	0.020–0.200	0.017	0.510
	Control	0.060	0.061	−0.060–0.180	0.325	
T2 → T3 ER → PMD	Compassion	0.190	0.041	0.110–0.270	<0.001	0.090
	Control	0.080	0.051	−0.020–0.180	0.117	
T2 → T3 PMD → ER	Compassion	0.090	0.041	0.010–0.170	0.028	0.565
	Control	0.050	0.056	−0.060–0.160	0.371	
T3 → T4 ER → PMD	Compassion	0.150	0.051	0.050–0.250	0.003	0.267
	Control	0.070	0.051	−0.030–0.170	0.170	
T3 → T4 PMD → ER	Compassion	0.080	0.051	−0.020–0.180	0.117	0.648
	Control	0.040	0.071	−0.100–0.180	0.573	

β = standardized within-person cross-lagged coefficient; SE = standard error; CI = confidence interval. Wald *p* compares corresponding coefficients between conditions. ER = emotion regulation; PMD = prosocial moral development. The T1-to-T2 control ER-to-PMD *p* value is rounded to 0.050. All six between-condition Wald *p* values exceed 0.05.

Although the cross-lagged coefficients were numerically larger under compassion training, Wald tests did not detect a significant between-condition difference in any of the six parameters (*p* = 0.067–0.648; Table 12). The results therefore did not support H4’s prediction of stronger reciprocal within-person coupling under compassion training. The lack of a significant final-interval PMD-to-ER path also indicated that bidirectionality was not consistent across all intervals (Figure 6).

**Figure 6:**
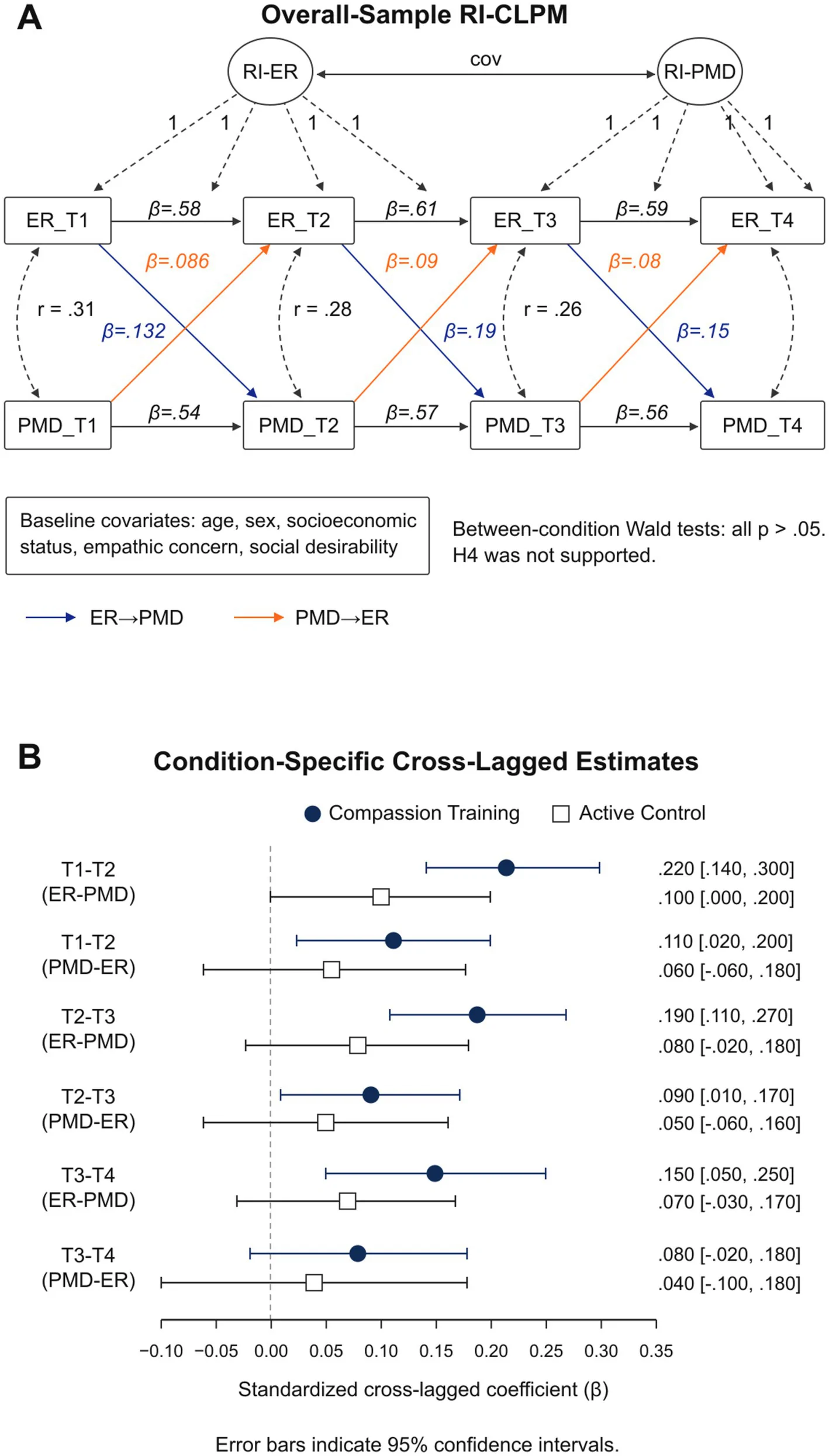
RI-CLPM estimates for emotion regulation (ER) and prosocial moral development (PMD). **(A)** Overall-sample model: χ^2^(48) = 102.3, CFI = 0.968, TLI = 0.954, RMSEA = 0.038 (90% CI 0.028–0.048), SRMR = 0.041. **(B)** Condition-specific estimates with 95% CIs. Between-condition Wald tests were nonsignificant (Table 12); H4 was not supported.

### 4.8 Sensitivity analyses

FIML and multiple-imputation point estimates were numerically similar, with absolute differences of 0.003–0.061 across the selected parameters (Table 13). Delta-adjusted sensitivity analyses yielded smaller Group × T4 estimates as δ decreased, but the estimates remained positive and statistically significant throughout the examined range (Table 14). For ER, the estimate decreased from 2.203 under MAR to 1.47 at δ = −0.75 SD (*p* = 0.014); for PMD, it decreased from 2.649 to 1.73 (*p* = 0.018). Thus, the direction and statistical significance of the 12-month differential changes were retained across these scenarios, while their magnitudes were sensitive to the assumed departure from MAR.

**Table 13:** Sensitivity analysis comparing FIML and multiple imputation results.

Parameter	FIML estimate	MI estimate	FIML − MI
Group × T2 (emotion regulation)	3.417	3.389	0.028
Group × T4 (emotion regulation)	2.203	2.156	0.047
Group × T2 (prosocial moral development)	4.216	4.174	0.042
Group × T4 (prosocial moral development)	2.649	2.588	0.061
ER → PMD cross-lagged path, T1 → T2 (β)	0.132	0.129	0.003
PMD → ER cross-lagged path, T1 → T2 (β)	0.086	0.081	0.005

Difference = FIML estimate − MI estimate. FIML = full-information maximum likelihood; MI = multiple imputation. Differences describe numerical agreement between point estimates.

**Table 14:** Delta-adjusted sensitivity analyses for 12-month differential change.

δ scenario	ER Group × T4	*p*	PMD Group × T4	*p*
MAR	2.203	<0.001	2.649	<0.001
−0.25 SD	2.04	<0.001	2.43	<0.001
−0.50 SD	1.81	0.002	2.14	0.001
−0.75 SD	1.47	0.014	1.73	0.018

MAR = missing at random. Negative δ scenarios represent progressively lower unobserved outcomes. Group × T4 estimates are differences between conditions in change from baseline to 12 months. Results concern the specified sensitivity scenarios.

## 5 Discussion

Among adolescents from four Chinese schools, compassion training produced greater baseline-to-wave change in ER and PMD than relaxation training, including at 12 months. The piecewise results indicated larger gains during the intervention followed by attenuation during follow-up, partially supporting H3. Within-person ER predicted subsequent PMD across all three intervals in the compassion condition, whereas the reverse association was significant in the first two intervals only. However, formal between-condition tests did not support H4. The distinction between an intervention effect on average outcomes and an intervention effect on reciprocal temporal coupling is central to interpreting these findings.

Comparison with published trials calls for outcome-specific interpretation. In the MYRIAD trial, 84 schools and 8,376 participants contributed to an analysis that found no superiority of universal school mindfulness over usual provision at one year (Kuyken et al., 2022). In a smaller active-control trial of 59 adolescents with subsyndromal depression, Bluth et al. (2024) reported a 2.6-fold higher risk of clinically significant depression in the control condition across 36 weeks, while self-compassion increased in both conditions and continuation-session attendance was poor. Xavier et al. (2023) found no significant group-by-time interaction for emotional control, empathy, or cooperation despite some within-group improvements. Differences in age, clinical risk, outcomes, comparator, and follow-up may contribute to this variation in findings.

Baseline-SD-standardized differential changes were approximately 0.49 for ER and 0.44 for PMD at post-test, and 0.32 and 0.28 at 12 months. These estimates describe the magnitude of the relative changes, which were smaller at later assessments. Their practical importance should be considered alongside classroom functioning, student engagement, and implementation burden. Matching contact time and prescribed home practice strengthened the comparison with relaxation training, although expectancy, facilitator allegiance, and actual practice could still influence outcomes. The decline after the intervention suggests that maintenance strategies, including booster sessions or continued practice support, merit evaluation.

The observed pattern may reflect a process through which improved emotion regulation supports more stable prosocial engagement. Developmental research indicates that empathy and related socioemotional processes are closely associated with prosocial behavior during childhood and adolescence (Qiu et al., 2024), while longitudinal evidence suggests that empathy and prosocial behavior can develop in interconnected ways over time (Carrizales et al., 2025). When confronted with interpersonal tension or another person’s suffering, adolescents who can identify, tolerate, and reappraise their own emotional responses may retain more attentional capacity for perspective-taking and other-oriented decision-making. Empathy has also been identified as an important pathway linking social influences with adolescent prosocial behavior (Li et al., 2025a). Under these circumstances, personal distress and self-focused rumination may become less dominant, allowing empathic concern to be translated more readily into moral intention or action.

The temporal pattern was compatible with a continuing contribution of ER to later PMD within the compassion condition. A reverse association was present across the first two intervals but not the final interval. Compassionate engagement may also support reappraisal of personal distress, a possibility consistent with associations involving interpersonal emotion regulation and self-compassion in adolescent studies (Mu et al., 2025; Nguyen et al., 2025). These cross-lagged associations do not establish causal mediation, and their differences across intervals may partly reflect unequal lag durations. Future studies could test whether changes in practice, classroom climate, or compassion-related processes mediate longer-term outcomes.

The analytical strategy distinguished differential average change, phase-specific growth, and within-person temporal association. Three-level models accounted for repeated observations within students and students within classes. Piecewise growth models distinguished intervention-period gains from follow-up trajectories. The RI-CLPM separated stable between-person differences from within-person deviations, avoiding their conflation in conventional cross-lagged models (Hamaker et al., 2015). Measurement-invariance results supported longitudinal and between-condition comparisons under the fitted models (Little, 2024). The active control, classroom randomization, and ITT analyses further strengthened the design.

The nonsignificant Wald tests are particularly relevant to H4. Significant associations within the compassion condition, combined with weaker or nonsignificant associations in the active-control condition, did not translate into significant differences between conditions. Formal parameter comparisons are essential for this distinction (Mulder and Hamaker, 2021). The results support temporal associations within the compassion condition but not an intervention-specific strengthening of reciprocal coupling; nonsignificant differences also do not establish equivalence.

Several limitations warrant consideration. Attrition reached 40.4% at 12 months, and completers differed in age, gender, and socioeconomic status. Delta-adjusted analyses retained positive differential changes over the examined range, but smaller estimates under more negative δ values show that effect magnitude depends on assumptions about unobserved outcomes. Scenarios outside this range could yield different conclusions. Self-reported outcomes may be influenced by social desirability and shared-method variance. The 34 randomized classes and four participating schools limit precision and generalizability, even with classroom-cluster adjustment. Facilitator blinding was infeasible, fidelity assessment covered only a sample of sessions, and individual practice adherence may have varied. Finally, the absence of systematic adverse-effect assessment limits conclusions about tolerability. Replication with stronger retention, additional schools, multimethod assessment, and prospective monitoring would address these limitations.

The exclusion of adolescents receiving clinical treatment or reporting a major traumatic event within the preceding six months limits generalizability to these groups. Future studies should evaluate the program in more inclusive school samples with appropriate clinical support.

The next step should be replication in a preregistered, multisite trial involving more schools, stronger follow-up procedures, and fully prespecified longitudinal-model constraints. The need for replication is especially relevant because recent reviews of compassion-focused interventions with children and adolescents indicate promising outcomes but also substantial heterogeneity and continuing limitations in the evidence base (Roberts et al., 2026). Adding brief booster sessions after T2 would be especially informative because this would allow researchers to test whether the observed decline reflects reduced practice and whether limited reinforcement can maintain the initial gains. Future assessments should include self-report, behavioral, observational, peer-reported, and teacher-reported indicators, together with systematic monitoring of possible adverse responses and implementation burden. Mechanism-focused analyses examining whether reappraisal, personal distress, empathic concern, classroom climate, or practice adherence account for later changes in prosocial moral development would also be valuable. Before subgroup findings inform implementation, heterogeneity by age, baseline regulatory capacity, socioeconomic context, and school environment should be examined in adequately powered samples.

The findings support further evaluation of compassion training as a classroom approach to ER and PMD in comparable settings. Maintaining gains after the program and identifying the processes responsible for change remain priorities for future trials.

## 6 Conclusion

In this cluster-randomized trial of 876 adolescents, compassion training produced greater improvement in ER and PMD than relaxation training, with relative advantages remaining at 12 months. Gains were concentrated during the intervention and attenuated during follow-up. H1 and H2 were supported, H3 was partially supported, and H4 was not supported: cross-lagged coefficients did not differ significantly between conditions. Positive 12-month differential changes persisted across the delta-adjusted sensitivity scenarios, although their magnitudes decreased under stronger departures from MAR. These findings support further evaluation of the curriculum while highlighting the importance of maintaining gains, improving retention, and testing generalizability across school settings.

## Data Availability

The raw data supporting the conclusions of this article will be made available by the authors, without undue reservation.
